# The Influence of Thermal Conditions on the Stability and Load-Carrying Capacity of Compressed Thin-Walled Composite Profiles

**DOI:** 10.3390/ma19061118

**Published:** 2026-03-13

**Authors:** Hubert Debski, Patryk Rozylo, Michal Kuciej, Katarzyna Falkowicz, Pawel Wysmulski, Adam Tomczyk, Przemyslaw Mazurek

**Affiliations:** 1 Department of Machine Design and Mechatronics, Lublin University of Technology, Nadbystrzycka 36, 20-618 Lublin, Poland; h.debski@pollub.pl (H.D.); p.rozylo@pollub.pl (P.R.); 2 Department of Mechanics and Applied Computer Science, Bialystok University of Technology, Wiejska 45C, 15-351 Bialystok, Poland; m.kuciej@pb.edu.pl (M.K.); a.tomczyk@pb.edu.pl (A.T.); 3 Department of Aerospace Engineering, Rzeszow University of Technology, Powstancow Warszawy 8, 35-959 Rzeszow, Poland; pmazurek@prz.edu.pl

**Keywords:** temperature impact, thin-walled composite structures, buckling, postbuckling, load-carrying capacity

## Abstract

This paper presents experimental and numerical investigations on thin-walled carbon-epoxy composite structures subjected to axial compression under varying thermal conditions. The primary objective of the study was to determine the influence of temperature on the stability, postbuckling behavior, and load-carrying capacity of the tested profiles. To achieve this, an innovative research methodology combining laboratory experiments and numerical simulations was developed, enabling a comprehensive assessment of the performance of compressed composite structures at different operating temperatures. The obtained results allowed for both qualitative and quantitative evaluation of the temperature-dependent behavior (from −20 °C to +80 °C) of thin-walled composite elements under compressive loading, offering new insights into their structural performance in thermally variable environments. The maximum percentage change in load capacity under variable thermal conditions was approximately 26.5%. At sub-zero temperatures (−20 °C), a slight effect on the load-carrying capacity of composite structures was observed, with a change in stiffness of a few percent. At increased above-zero temperatures (+80 °C), a significant change in stiffness (up to several dozen percent) was observed. The strengths of the work are a relatively extensive experimental program across several temperatures and stacking sequence composites, the use of digital image correlation to capture buckling and postbuckling deformations, and the parallel use of numerical modeling.

## 1. Introduction

Continuous fiber-reinforced polymer composites are modern materials that are widely used in many branches of industry due to their mechanical properties. The use of these materials in modern aerospace or automotive structures primarily results from their high strength in relation to low density [1,2,3]. Very good mechanical properties of polymer composites are achieved through the use of modern fabrication methods, particularly autoclaving [4]. These materials belong to a group of thermoset composites that acquire their final properties through fabrication at high temperatures, which causes the resin (which is the binder of a composite material) to undergo polymerization. Materials produced in this way are thin-walled structures known as laminates, and they are used as structural elements in load-carrying systems. One example of this type of elements are thin-walled profiles with different cross-sectional shapes that are used as load-carrying components for reinforcing thin skin panels [5,6,7].

As previously mentioned, thin-walled composite components used in the design of aircraft or vehicles are exposed to variable operating conditions, particularly varying temperature that can significantly affect the characteristics and properties of these materials [8,9,10,11,12,13]. This is particularly true of thin-walled structural members which, due to their design, may undergo buckling under certain loads [14,15,16]. The buckling of a load-carrying structural component can lead to either complete or partial loss of its capacity to continue carry loads, which can have a significant impact on the operation of the structure with further loading [17,18,19,20]. In light of the above, the determination of the effect of operational factors on the variations in material characteristics causing a significant change in the behavior of a structural element becomes important. Previous research on thin-walled composite elements has primarily focused on analyzing their behavior under mechanical loading at room temperature conditions. The literature extensively discusses the mechanisms of buckling in thin-walled profiles with various cross-sectional geometries, using both experimental and numerical approaches. Particular attention is given to local and global buckling phenomena as well as the onset of damage in composite laminates. Nonetheless, many existing analyses are limited to geometrically simple specimens or fail to consider realistic operational environments, such as variable thermal exposure during service. In response to this research gap, in the present study, the impact of temperature on the buckling of thin-walled carbon-epoxy composite profiles under compressive load is investigated [13]. Experiments are conducted at different temperatures in order to determine whether and to what extent temperature affects the critical load and buckling mode of the profile.

While carbon fibers offer excellent high-temperature resistance, the overall thermal stability of CFRPs is primarily limited by the polymer matrix. As service temperatures approach the glass transition temperature (*T_g_*), a significant degradation of matrix-dominated properties—such as compression, shear, and interlamination strength—is observed, necessitating careful consideration of *T_g_* for structural applications [21].

The research presented represents an innovative contribution to existing investigation on the thermomechanical stability of composite profiles. The strengths of the work are a relatively extensive experimental program across several temperatures and stacking sequence laminates, the use of digital image correlation (DIC) to capture buckling and postbuckling deformations, and numerical modeling via finite element method (FEM). It should be emphasized that the presented research approach is superior in terms of experimental resolution, range of thermal conditions (negative temperatures) and relevance to specific design scenarios and fills a gap in the literature for this particular experimental-numerical program. The obtained results provide valuable insights into the behavior of such elements under real-life operational conditions and may contribute to further development and optimization of lightweight composite structural components.

## 2. Materials and Specimen Geometry

**Figure 1:**
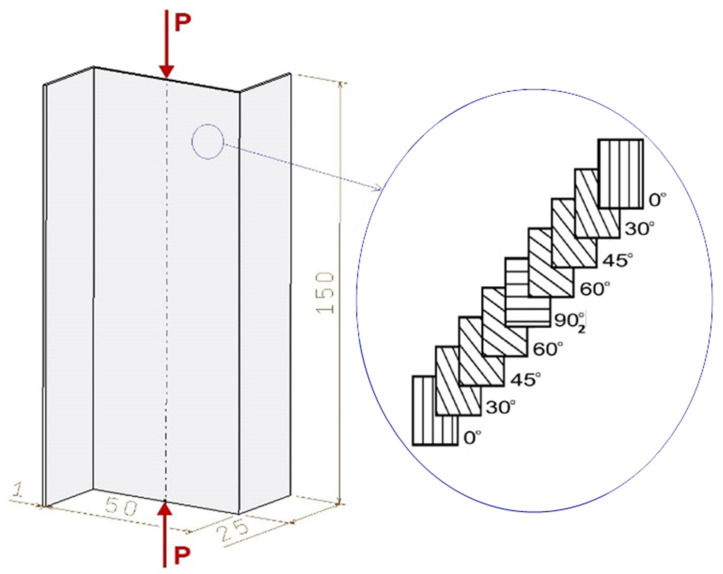
Geometric parameters of a composite Z-section profile with an example of a stacking sequence.

The study was conducted on thin-walled carbon–epoxy composite profiles that were fabricated by autoclaving. The profiles were made of a unidirectional pre-preg strip (Gurit^®^ EP 137: T_g_ 90 °C ÷ 100 °C), with different stacking sequences of individual plies, where the fiber orientation angle was defined relative to the longitudinal axis of the profile. The tested Z-section profiles had 10 plies symmetric to the mid-plane of the laminate, with the following stacking sequences: P1-[0/30/45/60/90]_s_, P2-[90/60/45/30/0]_s_, P3-[90/0/0/45/−45]_s_, P4-[45/−45/0/0/90]_s_, and P5-[0/0/45/−45/90]_s_. Each Z-section profile had a length of 150 mm, cross section of 25 × 50 mm, and a total wall thickness of 1 mm (single-ply thickness was 0.1 mm)—Figure 1.

## 3. Experimental Procedure

### 3.1. Test Setup

**Figure 2:**
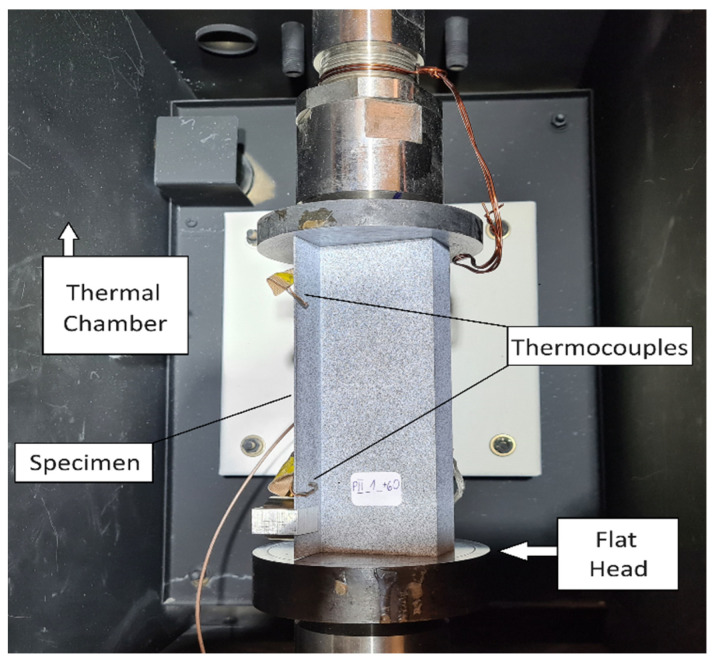
View of the experimental setup with a mounted specimen.

Experiments involved static compression of Z-section profiles using a universal testing machine, MTS 809 (Eden Prairie, MN, USA). Compression was applied by moving the upper crosshead with a constant speed of 1 mm/min. The specimens were simply supported at both ends, on round tables that were fixed in the grips of the testing machine. A schematic of the experimental setup is shown in Figure 2.

### 3.2. Temperature Conditioning

All experiments were conducted in a thermal chamber allowing the specimens to be compressed at different temperatures: −20 °C, room temperature of 20 °C, and elevated temperatures of 40 °C, 60 °C and 80 °C. Measurements at the negative temperature were made with the use of a cooling agent (nitrogen). Once the temperature in the chamber reached −20 °C, the specimen was maintained at this temperature for about 15 min prior to compression. This procedure made it possible to ensure the required temperature throughout the entire volume of the composite material. A similar procedure was followed for positive temperatures, with the specimens subjected to annealing for about 15 min at a given temperature.

The purpose of the study was to determine the effect of temperature on the buckling, postbuckling behavior and load-carrying capacity of axially compressed Z-section profiles. First of all, relationships were established between temperature and the buckling mode and critical load of the profiles. After that, an analysis was made of the postbuckling equilibrium paths of the compressed profiles, describing the relationship between the load and the deflection of selected points on the profile web, measured perpendicular to the web. Finally, the effect of temperature on the failure load of these profiles was determined. Results obtained for a profile compressed at a room temperature of 20 °C were taken as the reference level. The study is important especially in terms of the operation of composite structural components, as their properties may vary depending on the ambient temperature at which a given loaded component operates.

The problem of determining the critical load at which a compressed structural member undergoes buckling is of vital importance. Previous studies [22,23,24,25] have shown that the critical load of a real structure can be calculated by means of approximation methods. The aim of such calculation is to match the approximation function to the experimental results. It should be stressed that the correct selection of measurement points from all available measurement data is very important and has a decisive influence on final results. Related to this is the concept of so-called pivotal points, which was developed by Bronshtein and Semendyayev [26] and implemented by Spencer and Walker [27] for the analysis of experimental data for plates. According to this concept, the correct selection of pivotal points results in reasonably optimal fit of the expression under analysis to measurement data. During the tests, the specimens were prepared using a special matte paint that was resistant to temperature changes within the test range. The pattern used for digital image correlation (shown in dotted form) remained stable regardless of the variable temperature range.

### 3.3. Measurement System and DIC

**Figure 3:**
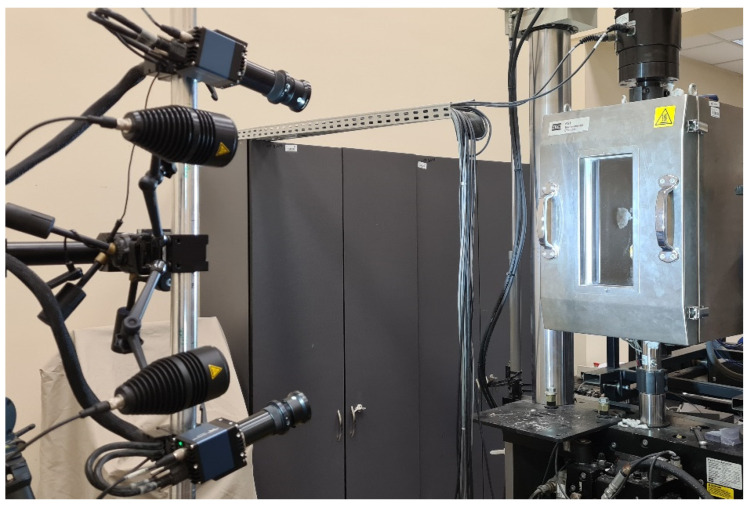
Digital measurement of deflection using the ARAMIS system.

In this study, the critical load was determined based on the experimental postbuckling path describing a load vs. deflection relationship, where the load value was read directly from the testing machine and the deflection was measured using the ARAMIS optical system. The operation of the ARAMIS measurement system is based on digital image correlation, which means that measurements are made in a non-contact manner and do not depend on the characteristics of tested materials [28,29]. The digital image correlation technique is based on two-image photogrammetry (stereophotogrammetry), which allows us to identify the location of individual points on the object in space. The ARAMIS optical measurement system uses Digital Image Correlation (DIC), which makes it possible to track changes in both point location and surface deformation. A definite advantage of the ARAMIS system is that it can perform measurements regardless of the geometry and temperature of tested objects. The ARAMIS system setup used in this study is shown in Figure 3.

### 3.4. Determination of Critical Load

**Figure 4:**
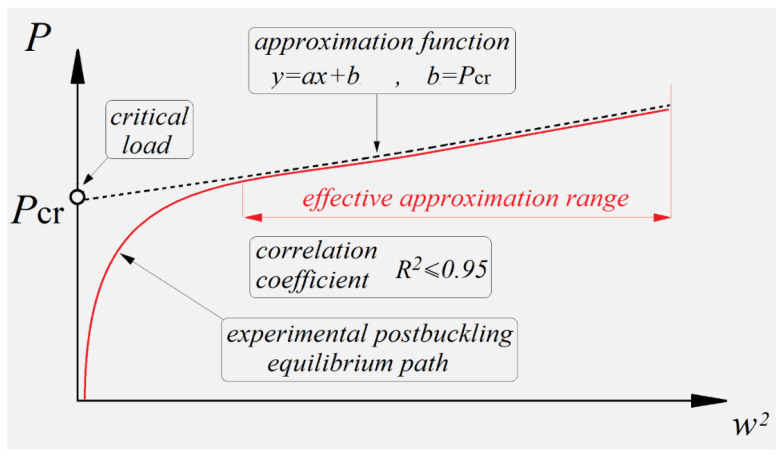
Schematic showing the procedure of critical load determination by *P-w^2^* method.

In this study, deflection was measured perpendicular to the web of a Z-section profile at points corresponding to the maximum amplitude of a buckling half-wave on the profile’s web. The postbuckling equilibrium path obtained thereby was then used to determine the approximate critical load of the profile by the *P-w*^2^ approximation method, as shown schematically in Figure 4.

Although in the *P-w*^2^ method the critical load is determined based on the postbuckling equilibrium path, the approximate critical load value is estimated based on the characteristics of the load versus the square of deflection perpendicular to the plane of the profile wall. The *P-w*^2^ postbuckling equilibrium path was approximated by a linear function of the form [22]:

(1) $$P=P_{cr}\frac{\alpha_{1}}{\alpha_{0}}w+\;P_{cr}$$

where α_0_, α_1_ are the unknown parameters of the function, *P* is the applied load, *P_cr_* is the unknown critical load, and *w^2^* is the square of deflection increase measured perpendicular to the profile wall. The critical load is defined as the point of intersection of approximation function (1) with the vertical axis of the coordinate system of the *P-w*^2^ postbuckling equilibrium plot. The accuracy of the applied approximation procedure depended on the correlation coefficient R^2^, with its value reflecting the level of agreement between the approximation function and the selected range of the approximated experimental curve—in other words, the higher the value of the correlation coefficient was, the more accurate the approximation process became. In this study, efforts were made to ensure that the correlation coefficient value was as high as R^2^ ≥ 0.95 [30].

## 4. Numerical Analysis

In addition to the experiments, the study also involved performing a FEM numerical analysis of the buckling of composite profiles under compression. The purpose of the numerical analysis was to determine the lowest buckling mode of the compressed profiles and the corresponding critical load. The critical load was determined using two independent methods: a linear calculation of an eigenvalue problem and a nonlinear calculation of the structure’s postbuckling equilibrium path describing a load vs. deflection relationship, with the deflection measured perpendicular to the web of the Z-section profile, at points of the highest value of a buckling half-wave.

In the solution of an eigenproblem that uses the principle of extremum potential energy, the equilibrium state of a structural system corresponds to the minimum potential energy [31,32]. This means that for stable systems, the second variation in potential energy must be positive. For such case, the solution of a stability problem involves solving the following equation [31,33]:

(2) $$\left(\left[K\right]+\lambda_{i}\;[S]\right)\;{\left\{\psi \right\}}_{i}=\left\{0\right\}$$

where [*K*] is the structural stiffness matrix, [*S*] is the stress stiffness matrix, *λ_i_* is the i-th eigenvalue, and *ψ_i_* is the i-th eigenvector of displacement. Here, the critical load is equal to the bifurcation load of an ideal structure, i.e., without pre-deflection.

The other procedure, which was specially developed to this end, makes it possible to determine the critical load of a predeflected structure based on the postbuckling path of load vs. deflection, with the deflection measured at the very same points as in the experiments (at the locus of the highest amplitude of a buckling half-wave on the Z-section profile web). The postbuckling characteristics of the structure were determined via a nonlinear numerical analysis for the solution of a stability problem by the incremental-iterative Newton–Raphson method [34]. Like in the experiments, the approximate critical load was estimated by the *P-w*^2^ approximation method.

**Figure 5:**
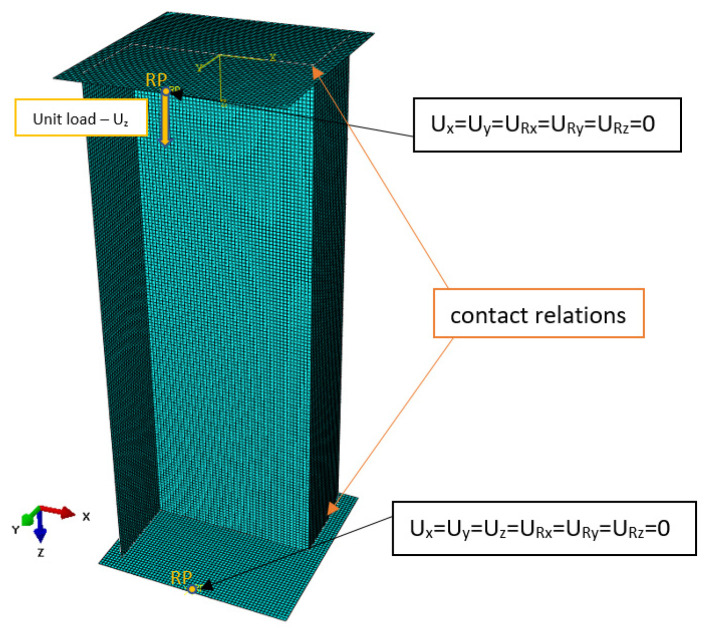
Discrete model of a Z-section profile.

The discrete model of the structure was designed using shell elements of the S4R type. The S4R finite element is a 4-node element with a first-order shape function, having six degrees of freedom at each node. The use of this type of finite element made it possible to define the structure and properties of the laminate with respect to finite element thickness. The developed discrete model consisted of 22,693 nodes and 22,200 finite elements, including 15,000 shell elements and 7200 rigid elements. A general view of the discrete model is shown in Figure 5.

The discrete model was designed using a model of orthotropic composite material in a plane state of stress, which was defined based on the experimentally determined mechanical properties of the composite [35,36]. Material testing was conducted at a room temperature of 20 °C and −20 °C, 40 °C, 60 °C, 80 °C. The temperature selection criteria were based on the fact that below −20 °C, the thermal chamber window would freeze over, making it impossible to record the behavior of the samples. At higher temperatures above +80 °C, the resin material would melt. The upper and lower thermal ranges were determined (based on preliminary experimental tests) by the aforementioned issue. For the test temperature of −20 °C, due to technical difficulties during the experiment and an insufficient number of valid specimens, only two mechanical properties—the in-plane Kirchhoff modulus and in-plane shear strength could be successfully determined. The mechanical properties of the composite material used in the numerical study are listed in Table 1.

Boundary conditions were defined by simple support of the Z-section profile ends on rigid plate elements. Contact relations between the profile ends and the plates were defined by a friction factor of 0.1. All translational and rotational degrees of freedom were constrained for the lower plate, while the upper plate was only allowed to move in the longitudinal direction of the profile, in order to enable compression of the structure. In the linear calculations (buckling analysis), the structure was loaded by a compressive force applied to the upper plate; in the nonlinear calculations, the structure was loaded via displacement of the upper plate in the longitudinal direction of the profile—Figure 5. The numerical calculations were performed for a profile compressed at a room temperature of 20 °C. The numerical analysis made it possible to validate the accuracy of the experimental approximation methods for estimating the critical load of a real structure.

## 5. Results—Structural Buckling Modes

### 5.1. Model Validation at Room Temperature

The obtained buckling mode of a compressed Z-section profile was local buckling of its web and walls, manifested by the formation of a given number of half-waves in the longitudinal direction of the profile. In this study, it was assumed that the basic, reference structural buckling mode would be that obtained for a case of a profile axially compressed at a room temperature of 20°C—the compression test results obtained at reduced and elevated temperatures for individual profiles were compared with these reference results.

**Figure 6:**
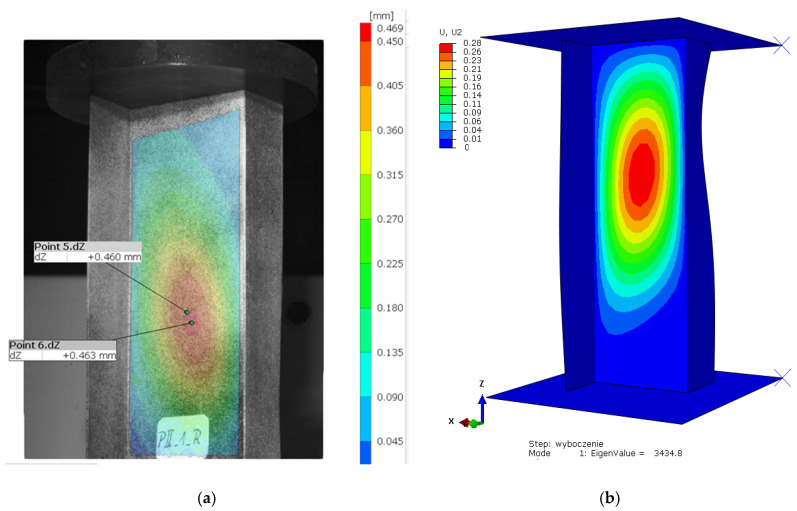
Buckling of a reference model (room temperature of 20 °C)—Z-section profile with the stacking sequence [0/30/45/60/90]_s_: (**a**) experimental results, (**b**) numerical results.

Figure 6 shows examples of the experimental (Figure 6a) and the numerical (linear eigenproblem solution—Figure 6b) buckling mode of a Z-section profile with the stacking sequence [0/30/45/60/90]_s_, corresponding to the lowest eigenvalue.

**Figure 7:**
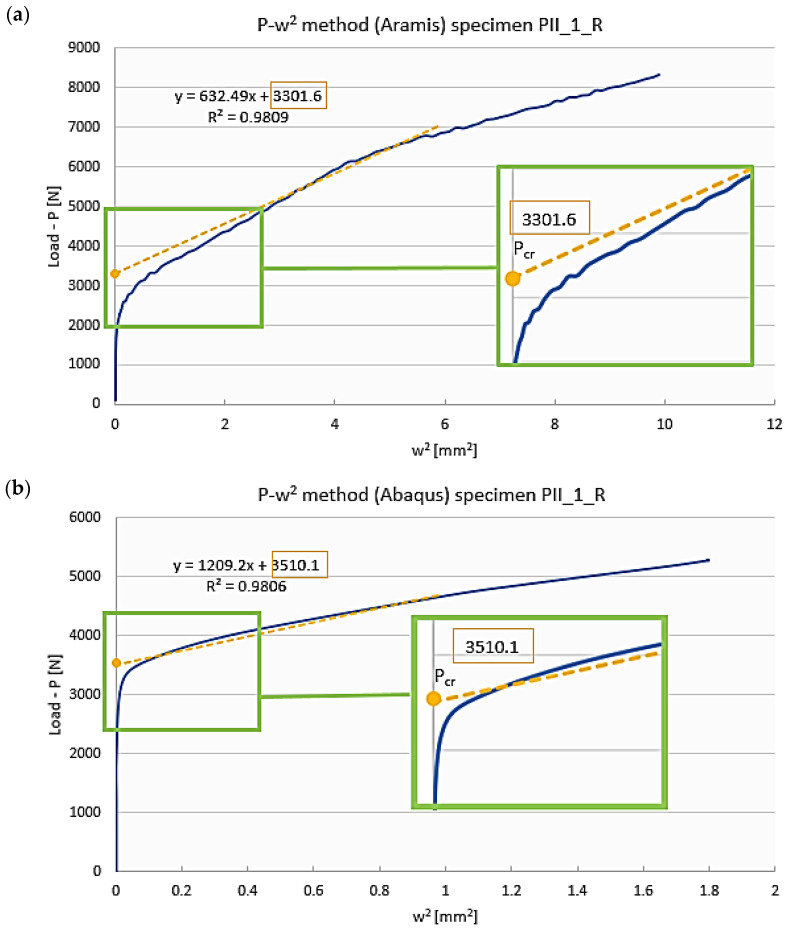
Procedure of critical load determination for a predeflected structure in the reference case (room temperature of 20 °C) of a Z-section profile with the stacking sequence [0/30/45/60/90]_s_: (**a**) experimental results—real structure, (**b**) numerical results—nonlinear calculations.

For this case, the buckling of a Z-section profile subjected to axial compression at the room temperature (reference model) was characterized by the formation of a single central half-wave on the web and walls of this profile. The buckling modes of the real structure and the numerical model show agreement—Figure 6. The qualitative agreement of the results made it possible to evaluate them in quantitative terms, including a comparison of the critical loads corresponding to the obtained buckling modes. The qualitative agreement was related to the similarity of shape, size and number of half-waves occurring within the buckling of the structure. The bifurcation load of the ideal structure obtained from the linear analysis of the eigenproblem, amounting to *P_cr_b_* = 3434.8 N (Figure 6b), was compared with the critical loads of the predeflected structure. This was done by determining the postbuckling equilibrium paths of load vs. square of deflection, with the deflection measured perpendicular to the web of the profile, at the point of the highest amplitude of half-wave deflection describing the profile’s buckling mode (Figure 6a). The critical load for the numerical model of a predeflected structure (nonlinear calculations) was determined by the *P-w*^2^ approximation method described in Section 2. A scheme illustrating the procedure for critical load determination is shown in Figure 7.

The critical load of a real structure was calculated based on the postbuckling equilibrium path determined with the Aramis system—the approximated critical load was *P_cr_EXP_* = 3301.6 N (Figure 7a). The numerical critical load was determined in the same way, using the postbuckling equilibrium path obtained from the nonlinear numerical calculations, where the approximated critical load was *P_cr___FEM_* = 3510.1 N (Figure 7b). A comparison of the obtained critical loads is given in Table 2.

Summing up the results obtained for the reference model of a Z-section profile with the stacking sequence [0/30/45/60/90]_s_, one can stress high agreement between the experimental and numerical critical loads. The maximum difference of 5.9% can be observed between the experimental critical load and the critical load of the predeflected structure. As for the numerical results, the difference between the critical load of the model with predeflection (nonlinear calculations) and the bifurcation load of the ideal model without geometric imperfection is 2.1%. The results thus show high agreement between the applied research procedures (primarily of the approximation method employed in the experiments), indicating their suitability for critical load determination of compressed composite structures.

### 5.2. Influence of Temperature on Buckling Mode

**Figure 8:**
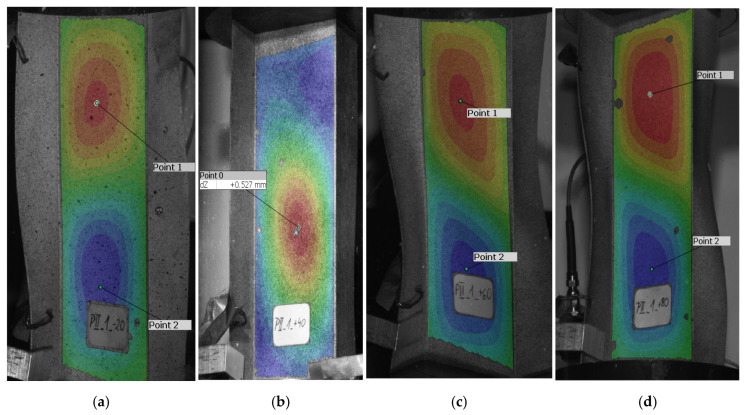
Buckling modes of a compressed Z-section profile with the stacking sequence [0/30/45/60/90]_s_—experimental results obtained at: (**a**) −20 °C, (**b**) +40 °C, (**c**) +60 °C, (**d**) +80 °C.

Proceeding in the same way as for the reference model, the critical load was also determined for real structures compressed at reduced and elevated temperatures. Figure 8 shows the buckling modes of a real structure, reflecting the lowest buckling modes of a compressed Z-profile with the stacking sequence [0/30/45/60/90]_s_.

An analysis of the buckling modes of a real structure reveals that the profile compressed at +40 °C (Figure 8b) is characterized by the formation of one central half-wave on its web and walls, which agrees with the buckling mode obtained for the reference model (Figure 6a). On the other hand, the buckling modes of the profiles compressed at −20 °C (Figure 8a), +60 °C (Figure 8c) and +80 °C (Figure 8d) are different—for these cases, structural buckling is manifested by the formation of two half-waves on the web and walls of the compressed profiles. This means that a change in the test temperature by ±40 °C and +60 °C relative to the reference model (+20 °C) caused a change in the buckling mode of the compressed profile, a phenomenon which was not observed when the temperature was changed by 20 °C (for the test conducted at +40 °C). In other words, the use of higher temperatures changes the buckling modes of the profile.

**Figure 9:**
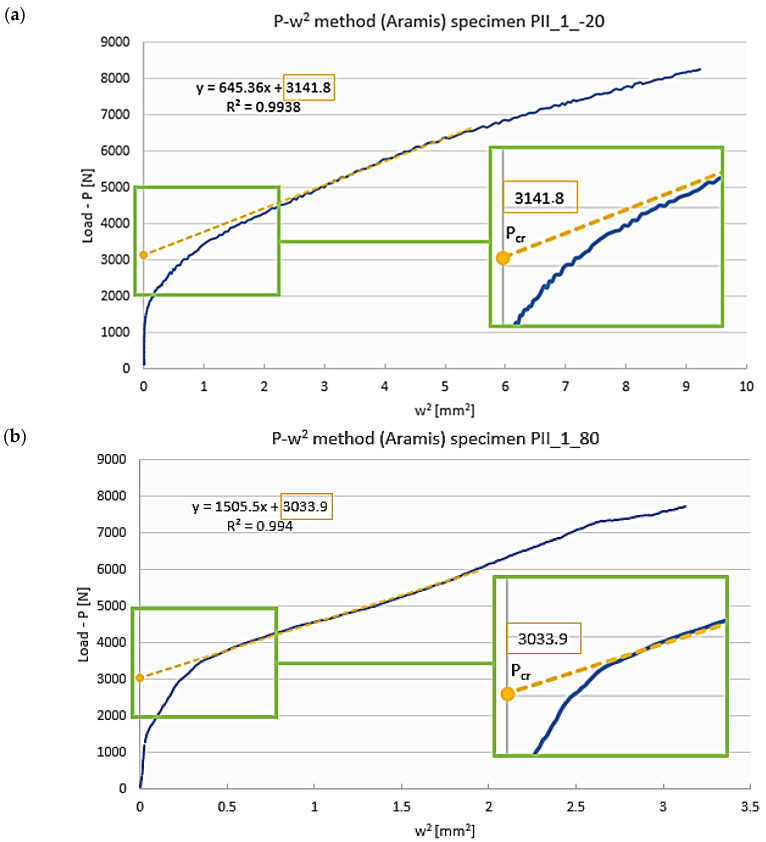
Procedure of critical load determination for a real structure (Z-section profile with the stacking sequence [0/30/45/60/90]_s_)—experimental results obtained at: (**a**) −20 °C, (**b**) +80 °C.

The experimental critical loads of the compressed profiles were determined in the same way as for the reference model, i.e., based on the postbuckling equilibrium paths of load versus square of deflection obtained by the *P-w*^2^ approximation method. This procedure of critical load determination for a Z-section profile with the stacking sequence [0/30/45/60/90]_s_ for extreme temperatures is shown in Figure 9.

### 5.3. Influence of Temperature and Layup on Critical Load

**Figure 10:**
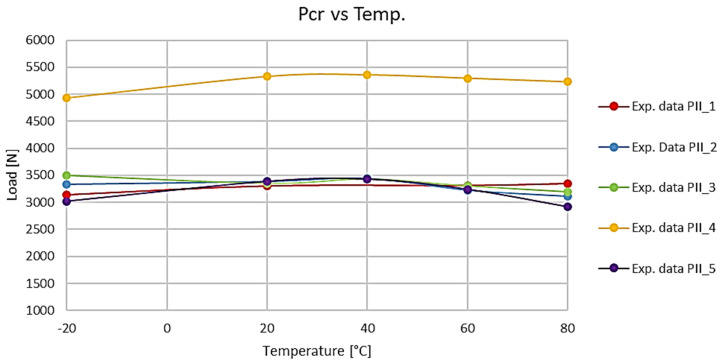
Critical loads of a Z-section profile for different ply layups and temperatures.

The experimental critical loads of a real structure determined with the above procedure for all tested ply layups in a Z-section profile for different temperatures are listed in Table 3 and plotted in Figure 10. The table does not give the numerical results for −20 °C due to the lack of material properties of this composite that were indispensable for performing the calculations.

The critical loads of the compressed Z-section profiles show that the operating temperature has a varying impact on the buckling of these structures, depending on the ply layup. The maximum differences in the buckling loads of real structures were observed for PII-5, where a 13.9% increase in the buckling load was observed between the reference model (+20 °C) and the experiment conducted at +80 °C. For other cases, the effect of the temperature on the buckling load was insignificant. This means that changes in the operating temperature within the tested range have a limited effect on the performance of these structures, which primarily refers to aerospace or automotive structural components that are usually made of the composite material under study. The experimental and numerical results showed high agreement—the maximum difference in the critical loads does not exceed 7.5% (PII-1), which confirms the accuracy of the developed discrete models.

## 6. Results—Postbuckling and Failure

### 6.1. The Effect of Temperature on Postbuckling Equilibrium Paths

**Figure 11:**
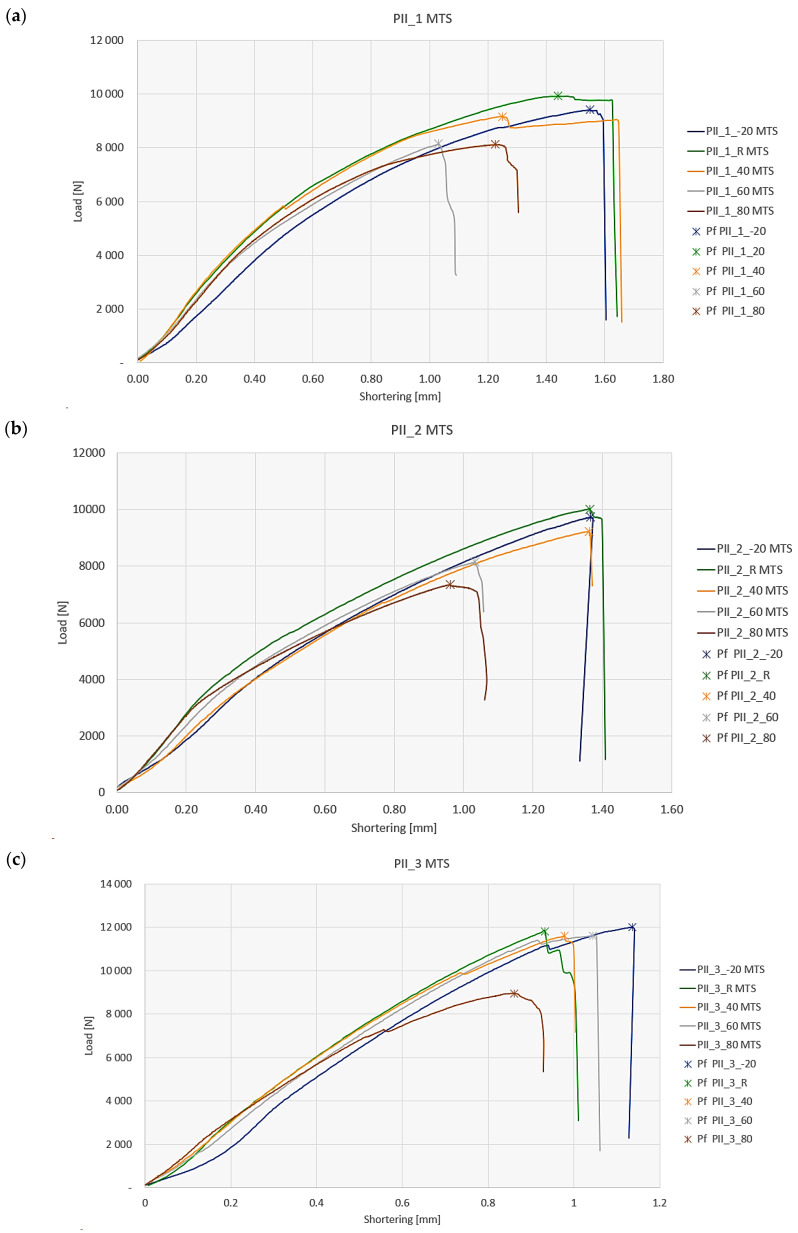

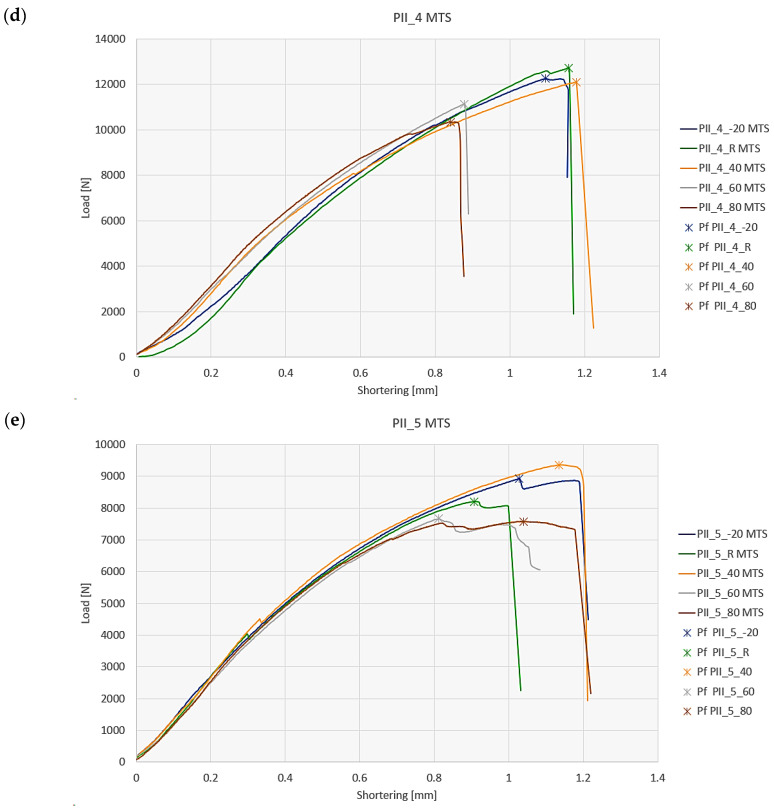
Postbuckling equilibrium paths of Z-section profiles under compression: (**a**) profile [0/30/45/60/90]_s_, (**b**) profile [90/60/45/30/0]_s_, (**c**) profile [90/0/0/45/−45]_s_, (**d**) profile [45/−45/0/0/90]_s_ (**e**) profile [0/0/45/−45/90]_s_.

The investigation of the postbuckling behavior of the compressed composite columns included analyzing the postbuckling equilibrium paths of real structures with the tested ply layups. To this end, a comparison was made of the postbuckling performance of the structures over the full load range depending on the temperature—Figure 11.

The postbuckling characteristics describing the performance of the composite profiles under the full load range make it possible to determine the failure loads of these structures under compression. The failure load values are marked by asterisks in Figure 11 and are listed in Table 4.

The experimental failure loads allow a quantitative evaluation of the effect of operating temperature on the loss of load-bearing capacity of the tested composite profiles. The last column (Table 4) shows the percentage difference between the failure load of a compressed profile at the reference temperature +20 °C and the failure loads obtained at the elevated and reduced temperatures, where the minus sign indicates a decrease and the plus sign an increase in the failure load value with respect to the reference temperature. With the exception of PII_5, the reduced temperature caused a decrease in the failure load by a maximum value of 5% (PII_3 showed a slight 1.7% increase in this load). On the other hand, an increase in the testing temperature led to a significant reduction in the failure load of about 20% and more at +80 °C depending on the ply layup. Somewhat different results were obtained for PII_5—for the reduced temperature the failure load increased by almost 9%, while at +80 °C the value of this load decreased by almost 8%. This difference in the behavior compared to the other tested composite layups may be due to the fact that in this particular layup, the two outermost plies on the inside and outside of the profile have fibers oriented in the 0/0 direction coinciding with the profile’s axis (axis of compression). For this case, the plies responsible for carrying the compressive load (fiber orientation coincided with the direction of load application) were the most exposed to the ambient temperature, which caused the material to behave differently from the other tested ply layups.

### 6.2. Influence of Temperature on Failure Mode

**Figure 12:**
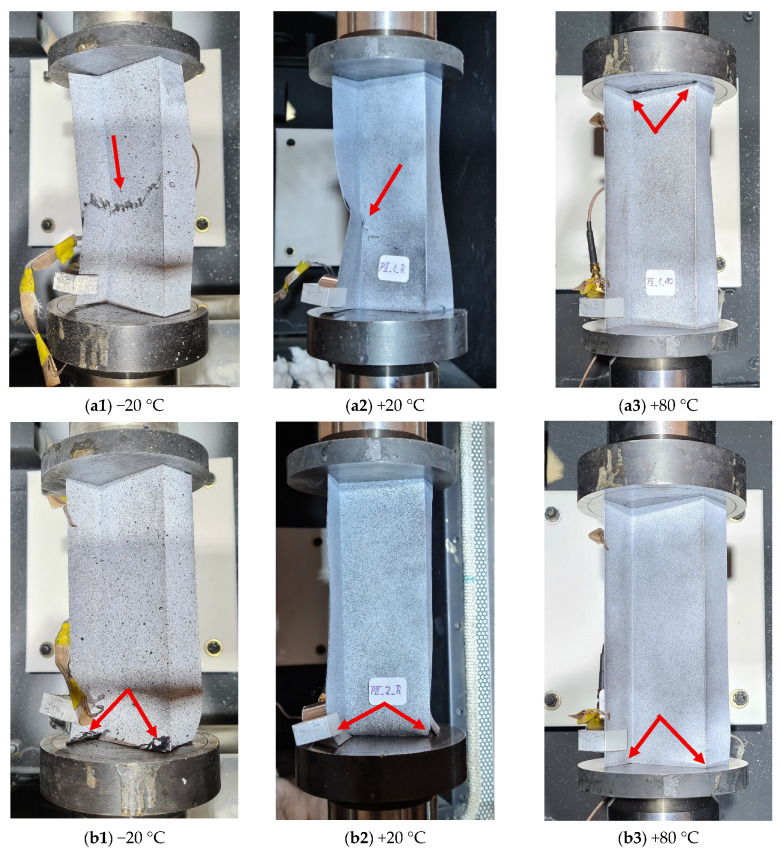

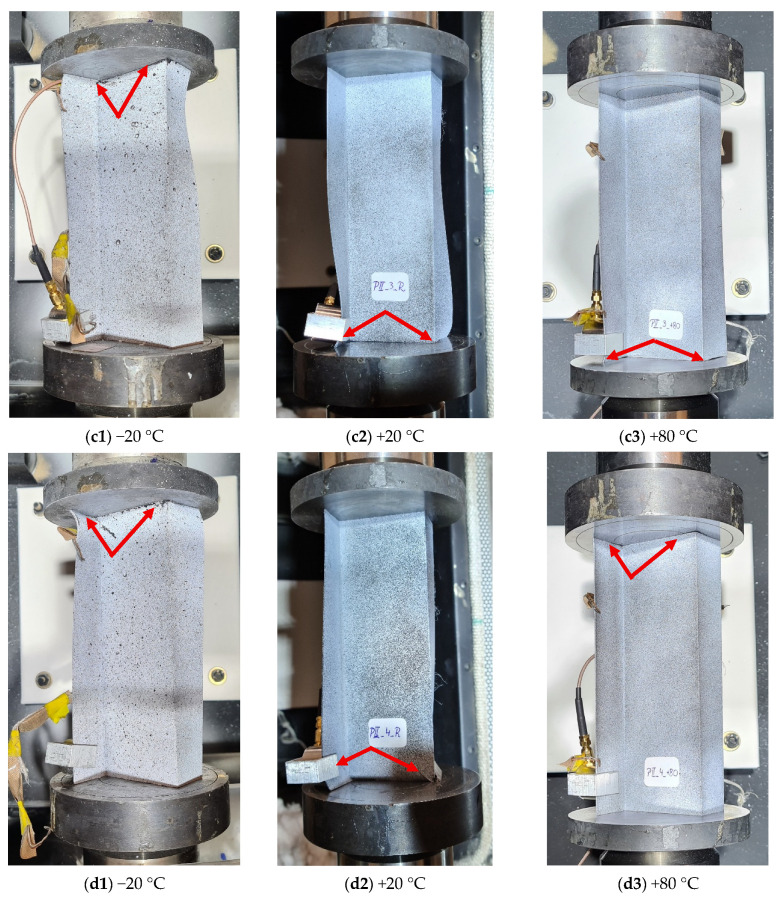

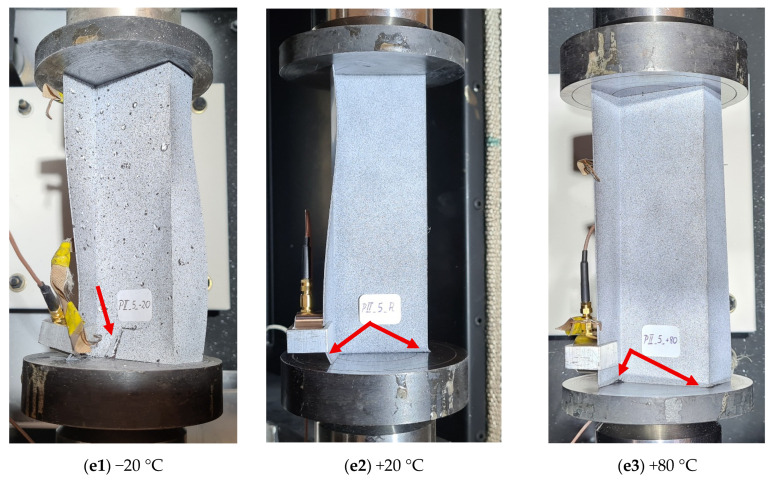
Failure modes of Z-section profiles under compression: (**a1**–**a3**) profile [0/30/45/60/90]_s_, (**b1**–**b3**) profile [90/60/45/30/0]_s_, (**c1**–**c3**) profile [90/0/0/45/−45]_s_, (**d1**–**d3**) profile [45/−45/0/0/90]_s_, (**e1**–**e3**) profile [0/0/45/−45/90]_s_.

Figure 12 shows the failure modes of individual composite profiles for different failure loads—owing to a significant number of results, the images show the results only for a reference temperature of +20 °C and two extreme temperatures of −20 °C and +80 °C, respectively, for each tested ply layup.

Whatever the ply layup, the failure modes of the tested profiles are the most extensive for the temperature 20 °C. This is due to increased brittleness of the composite resin resulting from the negative temperature; however, as shown in Table 4, it had no significant effect on reducing the failure load. At the highest temperature of +80 °C, the failure of individual profiles is hardly visible, which may result from high elasticity of the composite resin at the elevated temperature. For this case, however, a significant decrease in the failure load can be observed, ranging from 18.5 ÷ 26.5% depending on the ply layup. It should also be noted that the failure modes are locally concentrated; they are located mid-length or near the end sections of the profiles and are characterized by brittle fracture of the material and the occurrence of interply delamination.

## 7. Conclusions

This paper reported the results of a study investigating thin-walled composite Z-section profiles under compression. The primary objective of the study was to determine the effect of temperature on the stability of the tested structures, their postbuckling behavior and their load-carrying capacity under compression. To that end, an innovative research procedure combining experiments and numerical calculations was proposed in order to enable a comprehensive analysis of the stability and load-carrying capacity of the compressed structures under variable operating temperatures. The critical load of a real structure was determined by approximation methods first and then validated with numerical calculations by the finite element method. The numerical and experimental results showed high agreement, which confirmed the effectiveness of the employed approximation method for estimating the critical loads of a real structure. The results showed that the tested range of temperature had a varying effect on the critical load, depending on the ply layup—the maximum difference in critical loads was about 13.9% (PII_5), while for other cases these differences were considerably smaller.

Although the analysis of the postbuckling performance of the profiles showed no significant effect of the low operating temperature of −20 °C on the failure load, the observed zones of material failure were the most extensive for this case. On the other hand, the use of the highest tested temperature of +80 °C caused a significant decrease in the failure load ranging from 18.5 ÷ 26.5%, with the material failure zones being the smallest for this case. This can be attributed to high elasticity of the composite resin at considerably elevated temperatures.

The results made it possible to evaluate, in both qualitative and quantitative terms, the performance of the compressed thin-walled composite profiles depending on the operating temperature. This is especially important with respect to the design of thin-walled stiffening skins of load-bearing elements in aircraft or automotive structures. In this context, the experimental findings obtained for real structures are of crucial importance.

The research also observed that an increase in temperature (when the specimen was heated) had a significant impact on the reduction in composite profile stiffness. When the specimens were heated to higher temperatures (i.e., +60 °C and +80 °C), the composite material matrix softened. This resulted in a significant reduction in stiffness of the composite within the entire observed range. However, when the structures were kept at temperatures of −20 °C, 20 °C and 40 °C, it was found that the load-bearing capacity remained relatively consistent.

## Figures and Tables

**Table 1:** Mechanical properties of the tested composite material.

Temp.	Young’s Modulus		Poisson’s Ratio	Kirchhoff Modulus	Tensile Strength		Shear Strength	Compressive Strength	
T	E_1_	E_2_	ν_12_	G_12_	F_TU_ (0°)	F_TU_ (90°)	F_SU_ (45°)	F_CU_ (0°)	F_CU_ (90°)
°C	MPa	MPa	-	MPa	MPa	MPa	MPa	MPa	MPa
**−20**	-	-	-	5654.6	-	-	161.9	-	-
**20**	118,317.1	8339.4	0.31	4596	1781.4	49.1	137.5	707.6	115.5
**40**	120,981.4	7974.9	0.32	4346.1	1511.3	50.7	118.2	706.8	113.2
**60**	124,339.4	7323.2	0.34	3453.9	1387.3	49	96.1	484.8	77.8
**80**	127,962.3	5457.4	0.39	1908.1	1281.2	38.5	80.7	358.3	65.2

**Table 2:** Comparison of critical loads for the reference model.

Critical Load		
Critical Load [N] (Experimental)	Critical Load [N] (Nonlinear FEM)	Bifurcation Load [N] (Linear FEM Eigenproblem)
3301.6	3510.1	3434.8

**Table 3:** Comparison of critical loads—experimental and FEM numerical results.

Critical Load				
No.	Temp.	Critical Load [N] (Experimental)	Buckling Load [N] (Linear FEM)	Difference [%]
**PII_1**	−20	3141.0	---	---
	20	3301.6	3434.8	−4.56%
	40	3310.8	3558.3	−7.48%
	60	3343.7	3428.1	−2.52%
	80	3033.9	3054.3	−0.67%
**PII_2**	−20	3335.1	---	---
	20	3386.6	3286.3	2.96%
	40	3443.5	3374.2	2.01%
	60	3227.4	3252.9	−0.79%
	80	3115.9	2900.2	6.92%
**PII_3**	−20	3492.3	---	---
	20	3352.3	3352.9	−0.02%
	40	3427.9	3395.7	0.94%
	60	3306.2	3315.9	−0.29%
	80	3200.8	3108.4	2.90%
**PII_4**	−20	4937.8	---	---
	20	5326.2	5386.7	−1.14%
	40	5356.2	5449.1	−1.73%
	60	5294.9	5553.9	−4.89%
	80	5230.6	5335.6	−2.01%
**PII_5**	−20	3020.2	---	---
	20	3386.0	3389.3	−0.10%
	40	3429.1	3445.8	−0.49%
	60	3241.0	3259.9	−0.58%
	80	2916.0	2908.9	0.24%

**Table 4:** Comparison of experimental failure loads.

No.	Temp. [°C]	Failure Load [N] MTS	Failure Load [N] ARAMIS	TEMP. MTS/TEMP. REF. MTS
**PII_1**	−20	9410.03	9410.03	−5.17%
	20	9923.34	9923.38	0.00%
	40	9164.75	9164.78	−7.64%
	60	8145.22	8145.22	−17.92%
	80	8068.93	8068.92	−18.69%
**PII_2**	−20	9726.78	9726.78	−2.82%
	20	10,009.11	10,009.10	0.00%
	40	9219.77	9210.15	−7.89%
	60	8145.22	8145.22	−18.62%
	80	7347.76	7347.75	−26.59%
**PII_3**	−20	12,024.50	12,024.50	1.70%
	20	11,823.75	11,823.74	0.00%
	40	11,600.46	11,600.46	−1.89%
	60	11,603.99	11,603.99	−1.86%
	80	8960.90	8960.98	−24.21%
**PII_4**	−20	12,255.20	12,263.52	−3.64%
	20	12,718.55	12,718.57	0.00%
	40	12,104.77	12,096.04	−4.83%
	60	11,133.43	11,122.09	−12.46%
	80	10,343.31	10,355.83	−18.68%
**PII_5**	−20	8932.08	8950.18	8.89%
	20	8202.60	8204.52	0.00%
	40	9351.26	9360.88	14.00%
	60	7657.74	7642.34	−6.64%
	80	7568.22	7563.85	−7.73%

## Data Availability

The original contributions presented in this study are included in the article. Further inquiries can be directed to the corresponding authors.
